# *In vivo* CRISPR/Cas9-mediated gene integration corrects mucopolysaccharidosis type II in mice

**DOI:** 10.1016/j.gendis.2025.101928

**Published:** 2025-11-08

**Authors:** Hanfei Yu, Qian Qin, Shiyu Cui, Yujuan Wa, Kexian Dong, Wei Ji, Xueyuan Jia, Songbin Fu, Jie Wu, Wenjing Sun

**Affiliations:** a Department of Medical Genetics, School of Basic Medical Sciences, Harbin Medical University, Harbin, Heilongjiang 150081, China; b Key Laboratory of Preservation of Human Genetic Resources and Disease Control in China (Harbin Medical University), Ministry of Education, Heilongjiang 150081, China

Gene therapy is considered a promising method for treating monogenic diseases. Mucopolysaccharidosis type II (MPSII) is an X-linked recessive single-gene disease mainly caused by *IDS* mutations. Based on the CRISPR/Cas9 gene-editing tool, a dual adeno-associated virus (AAV) system was constructed, which functions through homologous independent targeting integration (HITI). One carried saCas9 with the liver-specific TBG promoter, and the other contained a small guide RNA (sgRNA) specific target to the first codon of the mouse *Alb* gene and carried the CDS of the human *IDS* gene. Two virus vectors mixed in a specific ratio were injected into *Ids*^X–/Y^ MPSII mice *via* the tail vein. The results showed that the expression of IDS in the liver tissue of treated MPSII mice was significantly higher than that of untreated MPSII mice. In addition, the IDS activity in gene-edited MPSII mice significantly increased, and the skeletal development of young mice also improved. The genome sequencing of the mouse liver confirmed that the human *IDS* donor sequence has been successfully inserted into the expected position of the mouse *Alb* gene. Our study provides an effective method for gene therapy of MPSII disease.

MPSII, also known as Hunter syndrome, is a rare monogenic disease caused by *IDS* gene mutation.^1^ *IDS* gene is located on human chromosome Xq28, containing 9 exons encoding 550 amino acids.^2^ All MPSII patients lack the enzyme iduronate-2-sulfatase (IDS), which leads to the accumulation of glycosaminoglycans (GAG) in the lysosome. With the accumulation of GAG, patients will experience a series of functional impairments. Due to the X-linked recessive inheritance of MPSII, it mainly affects males. It is estimated that one in every 100 000 male newborns is affected by MPSII.

At present, the treatment methods of MPSII mainly include enzyme replacement therapy, hematopoietic stem cell transplantation, and gene therapy. Enzyme replacement therapy for MPSII is simple and low risk, but it cannot completely cure the disease.^3^ Hematopoietic stem cell transplantation used for MPSII may not be perfect. When patients have intellectual disability or skeletal deformities, this method is difficult to alleviate intellectual and skeletal damage.^4^ In 2017, the candidate clinical trials (NCT03041324) for SB-913 *in vivo* genome editing obtained US FDA approval for the fast-track status of treating MPSII, which was developed by Sangamo Therapeutics, Inc. Unfortunately, due to the lack of observed clinical benefit, Sangamo decided to discontinue recruitment. In summary, further research is needed on the clinical treatment of MPSII to improve the accuracy and efficiency of addressing these challenging issues.

In this study, we employed *in vivo* genome editing to correct MPSII in preclinical research (Fig. 1A). To determine an efficient strategy for gene therapy of MPSII mice, a dual vector system of AAV was administered *via* tail vein injection. A preliminary assessment was conducted on the concentration and ratio of two viruses. MPSII mice received a tail vein injection of AAV.DJ-saCas9 (2 × 10^11^ GC), with AAV.DJ-Alb.sgRNA-hIDS-HITI (2 × 10^12^ GC), or the same dose of untargeted-donor AAV (Table S1). To evaluate the efficiency of the dual AAV vector system, the expression of IDS in liver tissue was first assessed. Six-week-old *Ids*^X–/Y^ MPSII mice were treated with the AAV dual vector system. After 7 months, the treated mice were euthanized, and quantitative reverse transcription PCR, immunoblotting, and immunofluorescence were performed to detect the expression of IDS in the liver. The results showed that the expression of IDS in the liver tissue of all treated MPSII mice (*n* = 3) was significantly higher than that of untreated MPSII mice, while the expression of albumin in the liver tissue was not affected (Fig. 1B–D).

**Figure 1:**
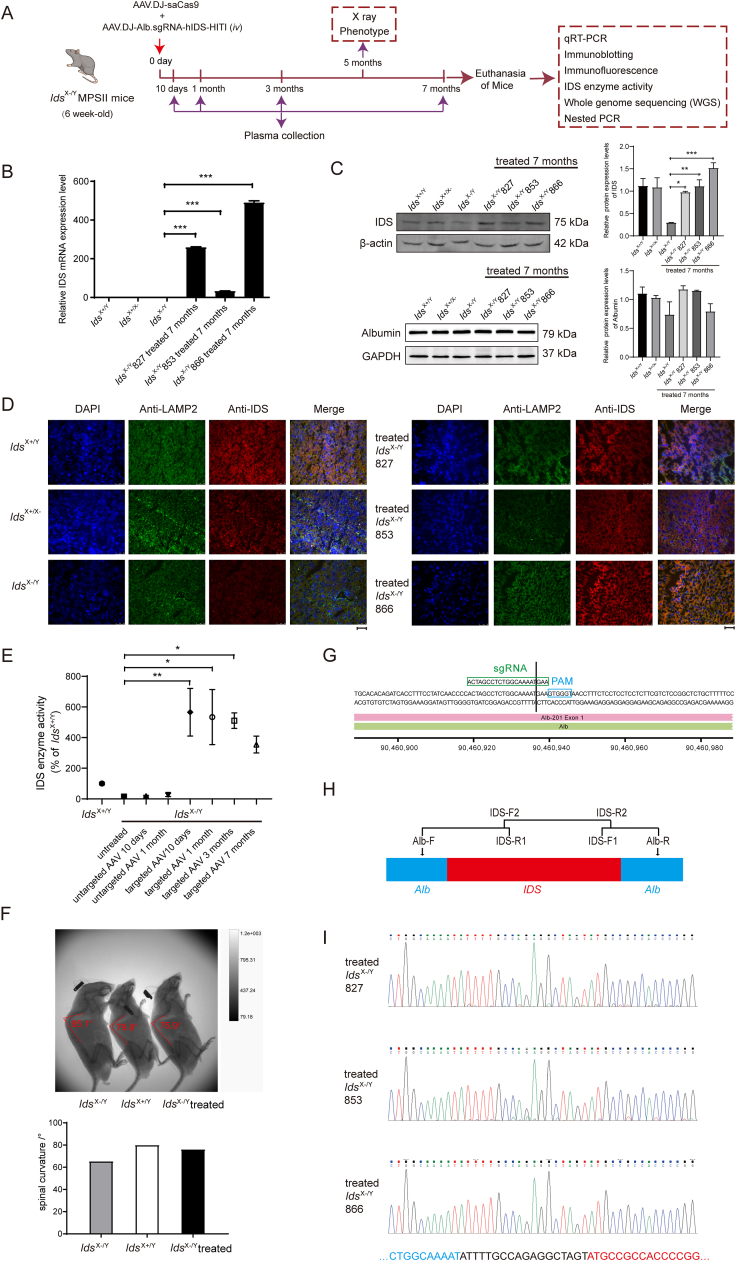
Homologous independent targeting integration (HITI)-mediated CRISPR/Cas9 gene therapy provides an effective model for the treatment of mucopolysaccharidosis type II (MPSII). AAV.DJ-saCas9 (2 × 10^11^ GC/pup) and AAV.DJ-Alb.sgRNA-hIDS-HITI (2 × 10^12^ GC/pup) were administered to 6-week-old MPSII mice *via* the tail vein. MPSII mice were euthanized 7 months after treatment (*n* = 3). The control-treated mice received AAV.DJ-saCas9 (2 × 10^11^ GC/pup) and AAV.DJ-hIDS (untargeted donor) (2 × 10^12^ GC/pup) *via* tail vein injection. **(A)** General schematic diagram. **(B)** Quantification of IDS mRNA in mouse liver by quantitative reverse transcription PCR. Data were shown as mean ± standard error of the mean. ∗∗∗*P* < 0.001, one-way *ANOVA* followed by *Tukey's* multiple comparisons test. **(C)** Immunoblotting with the antibodies against IDS and albumin on the liver tissue of wild-type (*Ids*^X+/Y^), MPSII heterozygous (*Ids*^X+/X–^), MPSII mice (*Ids*^X–/Y^), and MPSII mice treated with the dual AAV vectors for CRISPR/Cas9-mediated gene editing (*n* = 3). Data were shown as mean ± standard error of the mean. ∗*P* < 0.05, ∗∗*P* < 0.01, and ∗∗∗*P* < 0.001; one-way *ANOVA* followed by *Tukey's* multiple comparisons test. **(D)** Immunofluorescence staining with the antibodies against IDS on liver sections of MPSII mice treated with dual AAV vectors for CRISPR/Cas9-mediated gene editing. The stained areas typically represent the clusters of edited liver cells. The untreated controls show liver samples from the wild-type (*Ids*^X+/Y^), MPSII heterozygous (*Ids*^X+/X–^), and MPSII mice (*Ids*^X–/Y^). Scale bar, 50 μm. Double immunostaining against IDS (red) and lysosome marker LAMP2 (green) is presented. **(E)** The IDS enzyme activity in the plasma of MPSII mice was measured at 10 days, 1 month, 3 months, and 7 months after the dual AAV vector treatment. The IDS enzyme activity was also detected in wild-type (*Ids*^X+/Y^, *n* = 3), MPSII mice (*Ids*^X–/Y^, *n* = 3), and control-treated mice (*n* = 3). Data were shown as mean ± standard error of the mean. ∗*P* < 0.05 and ∗∗*P* < 0.01; one-way *ANOVA* followed by *Tukey's* multiple comparisons test. **(F)** X-ray images of mouse lateral views, from left to right, of untreated MPSII mice (*Ids*^X–/Y^), wild-type mice (*Ids*^X + Y^), and treated MPSII mice of the same age. **(G)** The cutting site diagram of AAV gene editing. The designed sgRNA sequence is ACTAGCCTCTGGCAAAATGAA, with the targeted cutting site located between the 2nd and the 3rd bases of the first codon of *Alb* gene. **(H)** DNA sequencing of gene-edited mouse liver. Primer sites are designed for nested PCR. The first round uses primers Alb-F and Alb-R; the second round uses primers Alb-F/IDS-R1, IDS-F2/IDS-R2, and IDS-F1/Alb-R. **(I)** Sanger sequencing results of the second round PCR products were aligned with the mouse *Alb* (blue) and human targeted *IDS* (red) genomic DNA sequences.

Our findings indicate that gene editing effectively triggers the expression of IDS in the mouse liver by driving the *Alb* promoter. The detection of IDS enzyme activity is of great significance in the diagnosis of MPSII.^5^ Plasma of the targeted treatment group was collected at 10 days, 1 month, 3 months, and 7 months after treatment, while plasma of the untargeted control group was collected at 10 days and 1 month after treatment. In addition, plasma samples were collected from wild-type mice and untreated MPSII mice. After IDS activity measurement, it was found that the plasma IDS activity of untreated MPSII mice (*n* = 3) was 15.19% of that of wild-type mice (*n* = 3). The plasma IDS activity of MPSII mice receiving untargeted AAV (*n* = 3) was 26.45% (10 days) and 18.58% (1 month) of the wild-type level. In contrast, the IDS activity of MPSII mice (*n* = 3) treated with the targeted AAV dual vector system was 565.86% (10 days), 533.82% (1 month), 510.65% (3 months), and 354.64% (7 months) of the wild-type level, respectively (Fig. 1E). A statistically significant difference was observed between gene-edited mice and untreated control mice through one-way *ANOVA* followed by *Tukey's* multiple comparisons test. Our data suggests that the dual AAV vector system based on CRISPR/Cas9 gene editing can significantly increase the plasma IDS activity of MPSII mice.

MPSII mice have less hair and a raised back, and some individuals even have difficulty moving. Six-week-old mice were treated with the dual AAV vector system for several months; phenotypic changes accompanied by increased IDS activity were observed. Compared with the control MPSII mice, the *IDS* gene-edited mice treated for 20 weeks had more hair with no serious back protrusion and were able to move normally (Fig. S1A and S1B). The results demonstrate that *IDS* gene editing can improve the phenotype of MPSII mice.

When MPSII mice were about four months old, they developed skeletal deformities. X-ray examinations on mice were performed to determine whether editing *IDS* gene at a young age improved skeletal development. It was found that the spine of MPSII mice was severely deformed at 26 weeks of age (65.1°). However, the spine (75.9°) of MPSII mice of the same age treated with the dual AAV vector system was close to that of wild-type mice (79.8°), indicating significant improvement in spinal development (Fig. 1F).

To accurately detect the location of gene editing, whole genome sequencing was performed on the liver tissues of all three *Ids*^X–/Y^ MPSII mice treated with the dual AAV vector system. Treated MPSII mice showed that approximately 1650 bp of the CDS fragment of the human *IDS* gene was inserted into the mouse chromosome 5 in liver tissue (Table S2). The off-target effects detected by whole genome sequencing in treated MPSII mice are detailed in Table S3. Clinically, no adverse effects were observed in these animals. We suppose that this may be attributed to the following factors: i) Most off-target events in intergenic regions minimize functional disruption; ii) The liver-specific TBG promoter restricts Cas9 expression primarily to hepatocytes, thereby reducing systemic exposure; iii) The observed off-target effects show low sequencing read counts, indicating limited biological impact.

To further elucidate the target sequence, nested PCR was performed. The results showed that the targeted *IDS* sequence was successfully inserted into *Alb* gene in all treated MPSII mice (Fig. 1G–I). Between the 2nd and 3rd bases of the first codon (atg) of *Alb* gene, which was the targeted cutting position, a sequence (5′-ATTTTGCCAGAGGCTAGT-3′) was detected in all treated MPSII mice before the targeted *IDS* sequence. This sequence is the reverse complementary sequence of the first 18 bases of sgRNA (5'-ACTAGCCTCTGGCAAAATGAA-3'). The theoretical cleavage position is between the third to last base and the fourth to last base of sgRNA, directly corresponding to the 18th and 19th bases of sgRNA in this study. In the inserted IDS sequence, a mutation from C to *G* (*IDS*: c.90C > G) was detected, which is a synonymous mutation (p.A30 = ). To summarize, our work demonstrates that the HITI strategy mediated by the CRISPR-Cas9 dual AAV system leads to therapeutic efficacy in the MPSII mouse model, thereby establishing a robust foundation for the development of innovative gene therapies for MPSII and other genetic disorders.

## CRediT authorship contribution statement

**Hanfei Yu:** Writing – review & editing, Writing – original draft, Visualization, Validation, Software, Methodology, Formal analysis, Data curation, Conceptualization. **Qian Qin:** Writing – review & editing, Writing – original draft, Visualization, Validation, Software, Methodology, Formal analysis, Data curation, Conceptualization. **Shiyu Cui:** Methodology, Formal analysis, Data curation. **Yujuan Wa:** Validation, Methodology, Data curation. **Kexian Dong:** Visualization, Methodology, Data curation. **Wei Ji:** Methodology. **Xueyuan Jia:** Methodology, Data curation. **Songbin Fu:** Writing – review & editing, Funding acquisition, Conceptualization. **Jie Wu:** Writing – review & editing, Conceptualization. **Wenjing Sun:** Writing – review & editing, Visualization, Funding acquisition, Data curation, Conceptualization.

## Ethics declaration

All of the animal studies were approved by the Ethics Committee of Harbin Medical University (HMUIRB20200006).

## Funding

This work was supported by the 10.13039/501100012166National Key Research and Development Program of China (No. 2021YFC2701002, 2016YFC1000504) and 10.13039/501100005046Heilongjiang Provincial Natural Science Foundation of China (No. JQ2022C004).

## Conflict of interests

The authors declared no competing interests.

## References

[1] Condò I. (2022). Rare monogenic diseases: molecular pathophysiology and novel therapies. Int J Mol Sci.

[2] Verma S., Pantoom S., Petters J., Pandey A.K., Hermann A., Lukas J. (2021). A molecular genetics view on mucopolysaccharidosis Type II. Mutat Res Rev Mutat Res.

[3] Chan M.Y., Nelson A.J., Ngu L.H. (2023). Long-term experience with idursulfase beta (Hunterase) in two adolescent patients with MPS II: a case series. Mol Genet Metab Rep.

[4] Selvanathan A., Ellaway C., Wilson C., Owens P., Shaw P.J., Bhattacharya K. (2018). Effectiveness of early hematopoietic stem cell transplantation in preventing neurocognitive decline in mucopolysaccharidosis type II: a case series. JIMD Rep.

[5] Zhong L., Gao X., Wang Y. (2023). Clinical characteristics and genotypes of 201 patients with mucopolysaccharidosis type II in China: a retrospective, observational study. Clin Genet.

