# Supplementary material for: In vivo CRISPR/Cas9-mediated gene integration corrects mucopolysaccharidosis type II in mice
Source: Genes Dis. 2025 Nov 8;13(3):101928. doi: 10.1016/j.gendis.2025.101928 (PMC12828814; doi:10.1016/j.gendis.2025.101928)
Supplement: Multimedia component 1 [file mmc1.docx]

**Supplementary data**

**Materials and Methods**

**Gene editing vector design and virus preparation**

For targeted knock-in, an AAV dual-vector system was employed in this study. The first vector, AAV.DJ-saCas9, expressed saCas9 under the control of a liver-specific TBG promoter. The second vector, AAV.DJ-Alb.sgRNA-hIDS-HITI, encoded a sgRNA targeting the initiation codon of the murine *Alb* gene (5′-ACTAGCCTCTGGCAAAATGAA-3′), designed using the GPP sgRNA Design Tool (https://portals.broadinstitute.org/gpp/public/analysis-tools/sgrna-design), and a HITI donor cassette containing the 1,653-bp CDS of the human *IDS* gene (NM_000202.5). The sgRNA specifically recognized the PAM sequence 5′-GTGGGT-3′ adjacent to the *Alb* start codon. saCas9-mediated cleavage occurred between the second and third bases of the Alb initiation codon (ATG), enabling targeted insertion of the hIDS CDS. This design facilitates transcription of the integrated human *IDS* gene under the endogenous murine *Alb* promoter. All AAV serotype DJ vectors were produced by Beijing Kerui Biological Technology Co., Ltd. (Beijing, China).

**MPSII mouse model**

Male *Ids* gene knockout mice modeling MPSII (B6N.Cg-ldstm1Muen/J, No.024744) and wild-type C57BL/6N controls were obtained from The Jackson Laboratory (Bar Harbor, ME, USA). All animals were housed under SPF conditions in the Animal Center Laboratory of Harbin Medical University. Experimental groups consisted of n = 3 mice per cohort, a sample size determined to maintain statistical rigor while adhering to the 3R principles (Replacement, Reduction, Refinement) of animal research ethics. All of the animal studies were approved by the ethics committee of Harbin Medical University (HMUIRB20200006).

**Tail vein injection of mice for gene therapy**

The mice were divided into four groups. The treatment group was MPSII mice (*Ids*^X-/Y^) injected with AAV.DJ-saCas9 and AAV.DJ-Alb.sgRNA-hIDS-HITI *via* tail vein for gene therapy. In addition, three control groups were: wild-type mice (*Ids*^X+/Y^), untreated MPSII mice (*Ids*^X-/Y^), and MPSII mice (*Ids*^X-/Y^) treated with AAV.DJ-saCas9 and AAV.DJ-hIDS (untargeted-donor *Ids*^X-/Y^).

The gene editing treatments were performed on 6-week-old male MPSII mice. Mice received a tail vein injection of AAV.DJ-saCas9 (2×10^11^GC) and AAV.DJ-Alb.sgRNA-hIDS-HITI (2×10^12^GC) or untargeted-donor AAV at the same doses. Plasma samples from the treatment group were collected at 10 days, 1 month, 3 months and 7 months after treatment, and plasma samples from the untargeted control group were collected at 10 days and 1 month after treatment. In addition, plasma samples were collected from wild-type mice and untreated MPSII mice. Animals were euthanized at 7 months after virus treatment, and liver tissue were collected for further experiments.

**IDS activity detection**

The plasma IDS enzyme activity was measured by the secondary incubation method of the fluorescent substrate. The assay was performed as follows: plasma 2 μL was diluted 1:5 in ultrapure water, then incubated with 20 μL 4-methylumbelliferone-α-L-pyranosuronic acid 2-sulfate disodium salt (1.25 mmol/L; Carbosynth #EM03201). After 4-hour incubation at 37°C, McIlvaine buffer (0.4 mol/L sodium phosphate, 0.2 mol/L citrate, pH 4.5) 40 μL and recombinant human alpha-L-Iduronidase/IDUA protein (1 μg/mL; R&D Systems #4119-GH) 10 μL were added to the reaction mixture. After 24-hour incubation at 37°C, the reaction was stopped by adding carbonate stop buffer (0.5 mol/L Na₂CO₃/NaHCO₃, pH 10.7) 200 μL. Fluorescence was measured using a fluorimeter (excitation 365 nm, emission 460 nm).

**qRT-PCR**

Total RNA was extracted from liver tissue using TRIzol™ Reagent (Invitrogen) and reverse-transcribed using the High-Capacity cDNA Reverse Transcription Kit (Roche, Basel, Switzerland). Quantitative real-time PCR was performed with LightCycler® 480 SYBR Green I Master (Roche) under the following cycling conditions: 95°C for 5 min; 45 cycles of 95°C for 10 sec, 60°C for 20 sec, 72°C for 20 sec. Primers used: hIDS: F: 5′-GAAAACATAACCCTGGCTCCTGA-3′, R: 5′-TTGTGTTGTGGGCTAGACGAAGAT-3′. Mus musculus Actb: F: 5′-CAGAAGGACTCCTATGTGG-3′, R: 5′-CATGATCTGGGTCATCTTTTC-3′. Expression was normalized to Actb using the 2^−ΔΔCt^ method.

**Western Blot**

Proteins were extracted in RIPA lysis buffer supplemented with cOmplete™ protease and PhosSTOP™ phosphatase inhibitors. Lysates (20 μg) were resolved on SDS-PAGE gels and transferred to PVDF membranes. After blocking with 5% non-fat milk/TBST, membranes were incubated overnight at 4°C with anti-IDS antibody (1:1000; Cloud-Clone Corp., #PAH833Hu01) or anti-Albumin antibody (1:2000; Abcam, #ab192603). Following secondary antibody incubation (1:5000; FITC-conjugated goat anti-rabbit IgG, Rockland #600-401-379 or HRP-conjugated goat anti-rabbit IgG, ZSGB-BIO #ZB-2301), signals were detected using an Odyssey Imaging System.

**Immunofluorescence of liver tissue samples**

Liver tissue from treated mice and control mice (*Ids*^X+/Y^ wild-type, *Ids*^X+/X-^ heterozygous, *Ids*^X-/Y^ MPSII mice, and *Ids*^X-/Y^ MPSII mice treated with the AAV dual vector system) were collected and sectioned at 5 μm thickness. Sections underwent fixation in ice-cold acetone (10 min), followed by 3×5-min PBS washes. Permeabilization was performed with 0.1% Triton X-100/PBS (4°C, 30 min). After additional PBS washes, non-specific binding was blocked with 4% BSA/PBS (37°C, 30 min). Primary antibodies of anti-LAMP2 [GL2A7] (1:200; Abcam #ab13524) or anti-IDS (1:500; Cloud-Clone #PAH833Hu01) were applied overnight at 4°C. Following 3×5-min PBS washes, sections were incubated with Alexa Fluor 568-conjugated goat anti-rabbit IgG (1:1000; Biotium #20098) for 1.5 hours at 37°C. Nuclei were counterstained with DAPI (1 μg/mL; 5 min). Images were obtained using the microscope Leica DMI 4000B (Leica Microsystems).

**On-target and off-target mutagenesis analyses of gene editing**

WGS of the treated mice on liver tissue was performed by Novogene Bioinformatics Technology Co. Lt (Beijing, China). The procedure is briefly described as follows. Genomic DNA extracted from the liver tissue was fragmented to approximately 350 bp by Covaris S220 sonicator and used to create a DNA library following established Illumina end-repair protocols. After determining the size distribution and concentration, the DNA library was sequenced on the Illumina Hiseq X platform for paired-end 150 bp reads. The raw image files were processed by base calling analysis. The high-quality sequences were compared to the mouse reference genome (UCSC: GRCm38) using Burrows-Wheeler Aligner (BWA) software.

For gene editing identification, genomic DNA from liver tissue of the *Ids*^X-/Y^ MPSII mouse and treated MPSII mice were extracted using QIAamp DNA Microkit from QIAGEN (Hilden, Germany) for PCR and Sanger sequencing. Nested PCR was performed targeting gene editing areas. Briefly, the genomic DNA was first amplified using the Alb-F primer (5′-ATGGCAAACATACGCAAGG-3′) and Alb-R primer (5′-ACCACCTAAGGGTTCTCAGA-3′) on the mouse *Alb* gene outside the region of *IDS* donor sequence, and the 2117 bp PCR product was purified by gel extraction. Then, the second round PCR were performed using the Alb-F primer and IDS-R1 primer (5′- CTGAAGGGGATGTGTGGCTT-3′) with the 914 bp product, IDS-F2 primer (5′-GCACCTTGCCTGACAAACAG-3′) and IDS-R2 primer (5′- TTGAAGCCAACCCACACAGT-3′) with the 937 bp product, IDS-F1 primer (5′-CCCTGGTAATCCCCGTGAAC -3′) and Alb-R primer with the product of 543 bp, which using the purified first round PCR amplicon as a template to detect whether *IDS* donor was inserted into mouse *Alb* gene. Sanger sequencing was performed on the second round of products, and then the sequencing results were compared with the fragment of design.

**Statistical analysis**

Treated and control groups were evaluated at least 3 male mice per group to ensure reproducibility. And the multiple group comparisons were analyzed using One-way *ANOVA* followed by *Tukey's* multiple comparisons test. Analyses were performed in GraphPad Prism 8.0 with significance thresholds defined as *P* < 0.05 (*), *P* < 0.01 (**) and *P* < 0.001 (***).

**Supplementary Figure 1. Phenotypic changes in MPSII mice treated with dual AAV system.** (A) Plan view of untreated MPSII mouse (*Ids*^X-/Y^), wild-type mouse (*Ids*^X+Y^) and dual AAV vector treated MPSII mouse. (B) Lateral view of untreated MPSII mouse (*Ids*^X-/Y^), wild-type mouse (*Ids*^X+Y^) and dual AAV vector treated MPSII mouse.


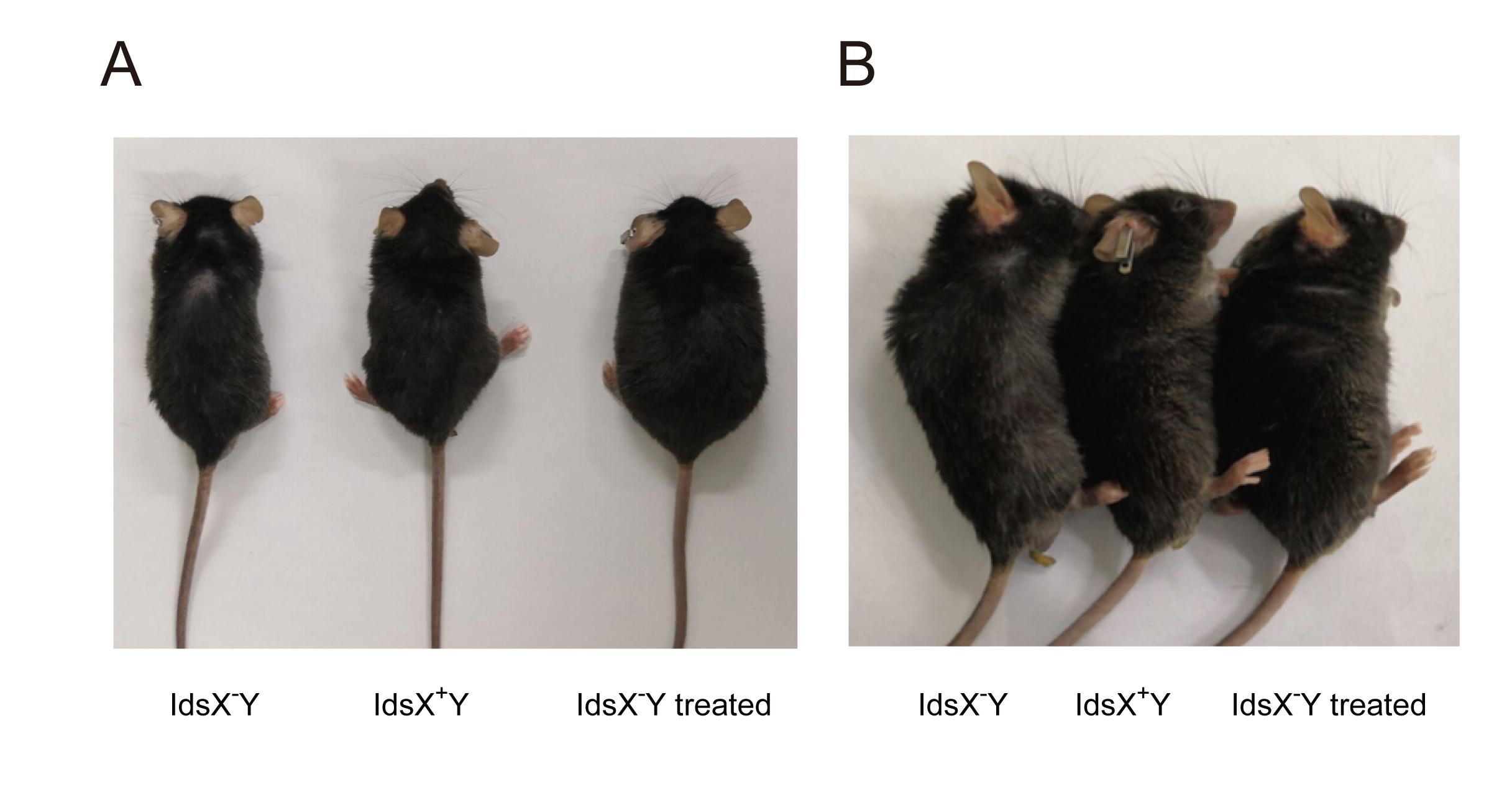


**Supplementary Table 1.** The strategy for MPSII mice gene therapy

| Mouse ID | Genotype | Age | Dosage of AAV.DJ-saCas9 (GC） | Dosage of AAV.DJ-Alb.sgRNA-hIDS-HITI (GC） |
| --- | --- | --- | --- | --- |
| 827 | *Ids*^X-/Y^ | 6 weeks | 2×10^11^ | 2×10^12^ |
| 853 | *Ids*^X-/Y^ | 6 weeks | 2×10^11^ | 2×10^12^ |
| 866 | *Ids*^X-/Y^ | 6 weeks | 2×10^11^ | 2×10^12^ |

**Supplementary Table 2.** The approximately inserted position of treated MPSII mice through WGS

| Mouse ID | Genotype | Chromosome | Start | End |
| --- | --- | --- | --- | --- |
| 827 | *Ids*^X-/Y^ | 5 | 90460918 | 90461019 |
| 853 | *Ids*^X-/Y^ | 5 | 90460808 | 90461024 |
| 866 | *Ids*^X-/Y^ | 5 | 90460806 | 90461021 |

**Supplementary Table 3.** The off-target effects of treated MPSII mice through WGS

| Mouse ID | Genotype | Chromosome | Start | End | Readsnum | Region |
| --- | --- | --- | --- | --- | --- | --- |
| 827 | *Ids*^X-/Y^ | 2 | 98666237 | 98667212 | 7 | intergenic region |
| 827 | *Ids*^X-/Y^ | 3 | 21984279 | 21984379 | 3 | intergenic region |
| 853 | *Ids*^X-/Y^ | 2 | 98665030 | 98667319 | 7 | intergenic region |
| 853 | *Ids*^X-/Y^ | 7 | 90441976 | 90442076 | 6 | intergenic region |
| 853 | *Ids*^X-/Y^ | 9 | 3000374 | 3000584 | 3 | intergenic region |
| 853 | *Ids*^X-/Y^ | 9 | 3020935 | 3024524 | 4 | intergenic region |
| 853 | *Ids*^X-/Y^ | 12 | 3109893 | 3110032 | 3 | intergenic region |
| 853 | *Ids*^X-/Y^ | 12 | 113091511 | 113091764 | 4 | intergenic region |
| 853 | *Ids*^X-/Y^ | 13 | 31419189 | 31419289 | 3 | intergenic region |
| 853 | *Ids*^X-/Y^ | 18 | 66201280 | 66201697 | 4 | *Ccbe1* |
| 866 | *Ids*^X-/Y^ | 2 | 98662287 | 98667173 | 10 | intergenic region |
| 866 | *Ids*^X-/Y^ | 7 | 84435960 | 84436060 | 3 | *Gm31284* |
| 866 | *Ids*^X-/Y^ | 9 | 3000496 | 3001474 | 4 | intergenic region |
| 866 | *Ids*^X-/Y^ | 9 | 122887121 | 122887221 | 3 | *Zkscan7* |
| 866 | *Ids*^X-/Y^ | 10 | 81590662 | 81590762 | 3 | *Tle2* |

**Author contributions**

HY, QQ, and WS were involved in all aspects of this study. HY, QQ, SC, YW, and KD conducted experiments. HY, QQ, SC, YW, WJ, and XJ performed WGS data analyses and sequencing analyses. HY and QQ did literature review and drafted this manuscript. WS, WJ, and SF critically reviewed this manuscript. All authors participated in manuscript formation by providing comments and suggestions. All authors read and approved of the final manuscript.
